# A comparison of feeding acetylated high-amylose maize starch and zinc oxide in weaned pigs experimentally inoculated with an enterotoxigenic strain of *Escherichia coli*

**DOI:** 10.1093/jas/skaf181

**Published:** 2025-05-24

**Authors:** Danica Evans, Bethany Bowring, Alison Collins, Julie Clarke, Jae-Cheol Kim, Josie Mansfield, John R Pluske

**Affiliations:** School of Agricultural Sciences, College of Environmental and Life Sciences, Murdoch University, Murdoch, Western Australia 6150, Australia; Elizabeth Macarthur Agricultural Institute, New South Wales Department of Primary Industries, Menangle, New South Wales 2568, Australia; Elizabeth Macarthur Agricultural Institute, New South Wales Department of Primary Industries, Menangle, New South Wales 2568, Australia; CSIRO Health and Biosecurity, Commonwealth Scientific and Industrial Research Organisation (CSIRO), Adelaide, South Australia, Australia; School of Agricultural Sciences, College of Environmental and Life Sciences, Murdoch University, Murdoch, Western Australia 6150, Australia; School of Agricultural Sciences, College of Environmental and Life Sciences, Murdoch University, Murdoch, Western Australia 6150, Australia; School of Agricultural Sciences, College of Environmental and Life Sciences, Murdoch University, Murdoch, Western Australia 6150, Australia

**Keywords:** acetate, diarrhea, gastrointestinal tract, performance, pigs, resistant starch, weaning, zinc oxide

## Abstract

Post-weaning diarrhea (PWD) remains a major problem for some pork producers, exacerbated by restrictions or bans on the use of antimicrobial compounds. Acetylated high-amylose maize starch (HAMSA) delivers acetate to the large bowel and may reduce the severity of enteric infections, including those caused by *Escherichia coli* (*E. coli*). This study examined the effects of HAMSA and zinc oxide (ZnO) supplementation on PWD and performance in pigs experimentally inoculated with an F4 enterotoxigenic strain of *E. coli* (F4-ETEC). Seventy-two weaned pigs were divided into three dietary groups: 1) control (no antimicrobial compounds); 2) control plus 3,000 mg ZnO/kg; and 3) control plus 50 g HAMSA/kg. Pigs commenced diets on the day of weaning, were inoculated with an F4-ETEC strain on days 5 and 6, and were fed diets ad libitum for 21 days. The incidence of PWD (χ^2^ = 0.035) and the diarrhea index (*P *= 0.032) were both lowest, commensurate with a lower plasma haptoglobin concentration (*P *= 0.010), in pigs fed ZnO than pigs fed other diets, despite there being a trend for an interaction (*P* = 0.088) in pigs fed HAMSA to have a lower F4 *E. coli*:total *E. coli* ratio on d 11 after weaning. Pigs fed ZnO and HAMSA grew faster (*P* = 0.009) and ate more (*P *= 0.048) in week 3 than control pigs. Overall, there was a trend (*P* = 0.065) for pigs fed the ZnO diet or HAMSA diet to eat ~ 20% more than those fed the control diet that resulted in a trend (*P* = 0.064) for ZnO- and HAMSA-fed pigs to weigh ~ 10% more than control-fed pigs at the end of the study. The HAMSA-fed pigs had a lower (*P *= 0.044) FCR in week 3, and overall (*P *= 0.003). Pigs fed HAMSA did not show any increase (*P *> 0.05) in their fecal short-chain fatty acid or acetate concentrations. The significant effect of HAMSA on FCR justifies further investigation as this may improve production efficiency in the post-weaning period following an enteric F4-ETEC infection.

## Teaser

Acetylated high-amylose maize starch (HAMSA) shows a reduced severity of infectious diarrhea in some species but has not been examined in newly weaned pigs. Pigs fed HAMSA and inoculated with an enterotoxigenic strain of *Escherichia coli* did not show reduced diarrhea relative to control and ZnO-fed pigs, but did show improved feed efficiency over the 21-d study.

## Introduction

Post-weaning diarrhea (PWD) caused by enterotoxigenic strains of F4 *Escherichia coli* (F4-ETEC) can remain a major problem in global pork production. Infection can cause a growth check in affected pigs, increased costs with prevention and treatment, and increased mortality in severe cases (Rhouma et al., 2017; Pluske et al., 2018; Laird et al., 2021; Canibe et al., 2022; Kim et al., 2022). In affected jurisdictions, restrictions or bans on the use of antibiotics and (or) pharmacological levels of Zn in diets have not reduced PWD on some farms, meaning that other dietary strategies, including supplementation with different types of dietary fibers, have been explored in attempts to mitigate the problem (Jha and Berrocoso, 2016; Flis et al., 2017; Canibe et al., 2022).

Resistant starch (RS) is a form of dietary fiber that passes largely undigested through the small intestine to the large bowel where it is fermented by the microbiota with the resultant production of short-chain fatty acids (SCFAs). Resistant starch can be chemically modified (type 4 RS) to deliver specific SCFA to the large bowel (Annison et al., 2003; Bajka et al., 2006), which in turn may positively influence gastrointestinal tract (GIT) function by promoting fluid and electrolyte uptake, regulating colonic muscular activity, and stimulating GIT immunity and microbial abundance and composition (Regassa and Nyachoti, 2018; Tan et al., 2021). Acetylated high-amylose maize starch (HAMSA) is a modified RS (Lim et al., 2015) that increases the concentration of acetate in the large bowel when ingested (Yap et al., 2021). In a mouse model, supplementation with HAMSA inhibited the translocation of *E. coli*-produced toxin to the bloodstream, protecting the mice from *E. coli*-induced death (Fukuda et al., 2011). Human patients with acute gastroenteritis experienced a reduced duration of diarrhea and tended to have lower fecal weights after receiving an oral rehydration solution supplemented with HAMSA (Pal et al., 2013). Chickens fed a diet supplemented with HAMSA had improved weight gain after challenge with *Clostridium perfringens* compared to the control group (M’Sadeq et al., 2015) and, in mice, feeding HAMSA protected against enteric infection with *Citrobacter rodentium* (Yap et al., 2021). These data suggest that RS4 in the form of HAMSA might have positive effects on the amelioration of enteric infections in monogastric animals, including post-weaned pigs.

The hypothesis examined in this experiment was that acetate delivered by HAMSA would reduce the incidence of PWD in pigs experimentally inoculated with F4-ETEC compared to pigs fed a control diet, with the incidence of PWD being comparable to pigs offered a pharmacological level of Zn. Other end points measured included the diarrhea index, production indices, fecal *E. coli* scores, fecal SCFA assessments, and selected bacterial ratios and counts.

## Materials and Methods

### Animal care

This study was reviewed and approved by the Animal Ethics Committee of Murdoch University (R2812/16). Animals were handled according to the *Australian code of practice for the care and use of animals for scientific purposes* (NHMRC, 2013).

### Animals, experimental design, diets, and housing

A total of 72 castrated male pigs (Large White × Landrace) weaned at approximately 21 days of age and weighing 6.1 ± 1.2 kg (mean ± standard error of the mean; SEM) was used. Pigs were obtained from a commercial pork producer in southwestern Australia on the day of weaning and transported to an experimental facility at Murdoch University. Pigs arrived in two batches that were 3 days apart but were placed on test according to the same experimental timeframe. Upon arrival, pigs were weighed, ear-tagged, and rectal swabs taken and cultured for baseline presence of β-hemolytic *E. coli*. The pigs were allocated to their experimental treatment group in six replicate pens of four pigs per pen according to a completely randomized block distribution (3 treatments × 6 replicate pens per treatment × 4 pigs per pen) and their live weight.

Pigs were fed one of three pelleted diets: 1) control (no antimicrobial compounds); 2) control with 3,000 mg ZnO/kg; and 3) control with 50 g HAMSA/kg (supplied by CSIRO Adelaide, Australia; manufactured by Ingredion, Bridgewater NJ, USA). The inclusion level was based on work in broilers showing an improvement in weight gain with HAMSA (M’Sadeq et al., 2015). The control diet was comprised primarily of wheat, soybean meal, barley, and dried whey, and was formulated to meet the animals’ requirements based on NRC (2012). Diet compositions and analyzed gross energy (GE) and nutrient contents are presented in Table 1. The diets, along with water, were offered on an ad libitum basis for 3 weeks after weaning.

**Table 1.** The composition of experimental diets (g/kg, as fed basis)

	Diet		
Ingredient	Control	ZnO	HAMSA
Barley	100	100	100
Wheat	447	441	385
Soybean meal	150	150	150
Bloodmeal	16.9	17.2	19.7
Fishmeal	79.8	80.4	86.3
Whey powder	158.7	158.7	158.7
Canola oil	29.7	31.6	33.5
L-Lysine	2.90	2.86	2.47
DL-Methionine	2.39	2.40	2.43
L-Threonine	1.36	1.35	1.25
L-Tryptophan	0.73	0.72	0.71
Vitamin/mineral premix^1^	1.5	1.5	1.5
Limestone	4.63	4.63	4.11
Dicalcium phosphate	2.25	2.21	1.83
Salt (NaCl)	2	2	2
Choline chloride (60%)	0.45	0.46	0.49
Zinc oxide	–	3	–
Acetylated high-amylose maize starch^2^	–	–	50
**Calculated composition (g/kg)**			
Digestible energy, MJ/kg	15.0	15.0	15.0
Protein	210	210	210
Fat	48.4	50.2	52.0
Neutral detergent fiber	89	88	82
Acid detergent fiber	27	27	26
Calcium	9	9	9
Digestible phosphorus	6.8	6.8	6.7
SID lysine	13.5	13.5	13.5
SID methionine + cystine	8.1	8.1	8.1
SID threonine	8.5	8.5	8.5
SID tryptophan	2.97	2.97	2.97
**Analyzed composition (g/kg)**			
DM	924	921	923
GE, MJ/kg	17.1	17.4	17.4
Crude protein	220	209	213
Crude fat	27	39	26
Starch	353	345	354
Neutral detergent fiber	84	90	96
Acid detergent fiber	34	26	32
Zinc, mg/kg	122	2,955	458

^1^Provided the following nutrients (per kg of air-dried diet): vitamins: A, 7,000 IU; D3, 1,400 IU; E, 20 mg; K, 1 mg; thiamine, 1 mg; riboflavin, 3 mg; pyridoxine, 1.5 mg; cyanocobalamin, 15 µg; calcium pantothenate, 10.7 mg; folic acid, 0.2 mg; niacin, 12 mg; biotin, 30 µg; minerals: Co, 0.2 mg (as cobalt sulfate); Cu, 10 mg (as copper sulfate); iodine, 0.5 mg (as potassium iodine); iron, 60 mg (as ferrous sulfate); Mn, 40 mg (as manganous oxide); Se, 0.3 mg (as sodium selenite); Zn, 100 mg (as zinc oxide).

^2^Acetylated high-amylose corn starch (HAMSA); product supplied by CSIRO, Adelaide, Australia.

Pigs were kept in pens of metal wire-meshed construction with plastic flooring and with a space allowance of at least 0.6 m^2^ per pig. Each pen was equipped with a nipple water drinker and a Rotecna TR5 ad libitum hopper (dimensions 75 × 30 × 68 cm) with five feeding spaces, with empty plastic containers provided for entertainment. Pens were housed in three different rooms, with two pens per treatment in each room. The ambient temperature was maintained at 28.0 ± 1.0° C (mean ± SEM). Pigs were monitored twice daily and weighed weekly. Feed residue from each pen was weighed weekly and feed wastage was recorded daily to calculate feed intake.

### Inoculation with enterotoxigenic *E. coli*, measurements of PWD, and fecal and blood sampling

Pigs were inoculated with an F4-ETEC (serotype O149:K88; toxins LT, ST, STb, EAST) on days 5 and 6 after weaning. Inoculation cultures of F4-ETEC were prepared as described previously (Heo et al., 2009). All pigs were orally dosed with the inoculum, using mild constraint, via a drench gun to provide ~ 9 mL aliquots of 1.03 × 10^9^ colony forming units (CFU)/mL of F4-ETEC per pig. Fecal rectal swabs were taken on days 0, 5, 7, 9, and 11 by inserting a cotton swab into the anus and were then inoculated on TSA with sheep blood agar plates (Thermo Scientific, Thebarton, Australia). Plates were incubated overnight at 37° C and assessed based on morphology and hemolysis. Scores were given on a scale from 0 to 5 according to the number of streaked sections containing viable hemolytic *E. coli*, where 0 was no growth and 5 was growth out in the fifth section of the plate (after Heo et al., 2009).

Fecal consistency and the incidence of diarrhea in individual pigs were visually assessed daily, by the same person at the same time each day, for 21 days after weaning. A score between 1 and 4 was given, as follows: 1) firm, well-formed feces; 2) soft-formed feces; 3) soft and loose shape; and 4) watery liquid consistency, with this considered as diarrhea. To allow for statistical analysis, the scores were converted into percentiles (1 = 0%, 2 = 33.33%, 3 = 66.7%, and 4 = 100%). The diarrhea index (DI) was calculated as the mean proportion of days pigs had diarrhea with respect to 14 days after weaning (after Heo et al., 2009). Pigs were monitored daily for signs of illness or injury and were monitored closely following experimental infection for signs of severe diarrhea. Pigs assessed as having severe diarrhea (score four feces) were treated intramuscularly with therapeutic antibiotics (Tribactral: triimethoprim and sulfadoxine, 1.5 mL/30 kg bodyweight daily; amoxicillin: amoxicillin trihydrate, 1 mL/20 kg bodyweight daily) as per veterinarian instruction.

Fecal samples were collected from six to seven individual pigs per treatment, on days 4 and 11 after weaning, for subsequent SCFA analysis and enumeration of specific bacterial groups by quantitative PCR (qPCR). Samples were stored at −20° C until analyzed.

Blood samples were collected (10 mL) from the two median weight pigs per pen (12 samples per treatment) on days 4 and d 11 after weaning via jugular vein puncture into a lithium heparin tube with a 20-gauge, 38 mm needle, and vacutainer. Lithium heparin tubes were immediately placed on ice. The heparin tube was centrifuged at 3,000 × *g* for 10 min at room temperature. Plasma was then collected and stored at −20° C until analyzed for plasma urea concentration, total antioxidant capacity (TAC), D-lactate, and the acute phase protein haptoglobin.

### Feed, digesta, and blood analyses

Diet samples were analyzed for dry matter (DM), GE, crude protein (CP), crude fiber, calcium, phosphorous, neutral detergent fiber (NDF), acid detergent fiber (ADF), starch, and Zn as per established protocols for InVivo Labs (Vietnam). The DM content was determined using method EC 152/2009. The nitrogen (N) content was determined using combustion method 2001.11 (AOAC, 2002) and CP content was calculated as N content × 6.25. The ADF and NDF contents were determined using ANKOM Technology methods 8 and 9, respectively (Ankom200 Fiber Analyzer, Ankom Technology, Macedon NY, USA). The GE content was determined using a ballistic bomb calorimeter (SANYO Gallenkamp, Loughborough, UK). Zinc content was determined using atomic absorption spectroscopy (AAS11 152/2009/EEC).

Fecal samples were analyzed for SCFA and pH content at CSIRO (Adelaide, Australia) using a modification of a previously published method (Bajka et al., 2006). Briefly, samples were weighed and mixed with three volumes of internal standard containing 1.68 mM-heptanoic acid (Sigma Chemical Co; pH 7.0), which was then homogenized. The pH was measured, and samples were then centrifuged at 2,000 × *g* for 15 min at room temperature. Supernatants were decanted and stored at − 20° C until analysis. Samples were thawed at 4° C, then centrifuged at 16,000 × *g* for 5 min at room temperature and acidified by the addition of 10 µL of 1 M phosphoric acid to 300 µL of supernatant. The samples were mixed thoroughly and filtered using Thomson Single StEP 0.45 µm PTFE Filter vials (Thomson Instrument Company, CA USA, 35540-100) before analysis using gas chromatography (6890N network GC system; Aligent Technologies Inc., Palo Alto, CA, USA).

The total esterified acetate concentration present in the diets was measured using a modification of a method by Clarke et al. (2011b). The HAMSA contained ~ 6% total acetate (J. Clarke, *pers. comm.*) and the diet had a total acetate concentration (before pelleting) of 172 µmol/g. Diet samples were diluted 1:3 (wt:volume) with internal standard (1.68 mM-heptanoic acid) and hydrolyzed by agitating for 2 h after addition of 3.75 times the sample volume 1.5 mol NaOH/L. Samples were then neutralized to pH 7.0, processed, and analyzed for SCFA by gas chromatography using the method described for fecal samples. The concentration of esterified acetate was highest in the HAMSA product, with 17 µmol/g of bound acetate lost during the pelleting process.

Plasma urea and haptoglobin contents were determined using a Beckman Coulter/Olympus Reagent Kit (OSR6134), and an in-house method NTM-62 based on Eckersall et al. (1999), respectively. All kits and methods were performed on an Olympus AU400 Clinical Chemistry Analyzer at the Department of Agriculture and Food, Animal Health Laboratories (South Perth, WA). The TAC and D-lactate assays were performed in-house using the OxiSelect Total Antioxidant Capacity Assay Kit (STA360), and Megazyme D-Lactic Acid (D-lactate) Assay Kit, according to the manufacturers’ instructions. The TAC assay was performed on an iMark Microplate Absorbance Reader (Bio-Rad, California, USA), and the D-lactate assay was performed on a Spark 10M multimode microplate reader (Tecan, Männedorf, Switzerland).

### DNA extraction and quantitative real-time PCR

The MagMAX Pathogen RNA/DNA Kit (ThermoFisher Scientific, Waltham, USA) was used to extract DNA from frozen fecal samples with DNA stored at −20° C until qPCR was performed. Quantitative PCR was used to measure the total counts of *E. coli*, *E. coli* with F4 fimbria, *Bifidobacterium* spp., and *Lactobacillus* spp. Primers and probes were synthesized by Biosearch Technologies (Novato, USA). TaqMan probes were synthesized with 5-carboxyfluorescein (FAM) on the 5’ end and Black Hole Quencher (BHQ-1) on the 3’ end. The specificity of primers and probes used was evaluated against reference 16S rRNA genes and 16S-23S rRNA intergenic spacer region sequences present in the National Center for Biotechnology Information (Bethesda, USA). Sequences were aligned using Clustal Omega (European Bioinformatics Institute, Saffron Walden, UK) and Sequencher (Gene Codes Corporation, New York, USA). The proposed primer and probe sequences were compared to known sequences to test for specificity using NCBI BLASTN 2.2.28 + (Zhang et al., 2000). Standard curves for each of the qPCRs were constructed in duplicate from 10-fold serial dilutions of each DNA extracted from known numbers of pure cultures of F4 *E. coli*, *E. coli*, *Bifidobacterium lactis*, and *Lactobacillus acidophilus* using the DNeasy Blood and Tissue Kit (Qiagen, Venlo, Netherlands). The primers and probes used are presented in Table 2.

**Table 2.** Primers and probes used for quantitative real-time PCR

Target	Primer/probe	Sequence	Reference
Total *E. coli*	Forward	CATTGACGTTACCCGCAGAAGAAGC	Bartosch et al. (2004)
	Reverse	CTCTACGAGACTCAAGCTTGC	
	Probe	ACGCAGGCGGTTTGTTAAG	Collins and Bowring (2023)
Lactobacilli	Forward	AGCAGTAGGGAATCTTCCA	Walter et al. (2001)
	Reverse	ATCGTATTACCGCGGCT	Modified from Heilig et al. (2002)
	Probe	CTGATGGAGCAACGCCGC	Modified from Delroisse et al. (2008)
Bifidobacteria	Forward	CGTCYGGTGTGAAAGTCCAT	
	Reverse	CTTCCCGATATCTACACATTCCA	Xiang et al. (2011)
	Probe	CGCTTAACGGTGGATCYGCGCC	
F4 fimbriae *E. coli*	Forward	GGTGATTTCAATGGTTCGGTC	Franklin et al. (1996)
	Reverse	ATTGCTACGTTCAGCGGAGCG	

One-tenth volume of the extracted DNA was added to each qPCR reaction. The F4 fimbria *E. coli* qPCR assay included 7.5 pmoles of both forward and reverse primers in the Bioline SensiMix SYBR Green Low-ROX reaction kit (Applied Biosystems, Foster City, USA). The product was amplified over 40 cycles of 95° C for 15 s, 65° C for 30 s, and 72° C for 60 s, following an initial denaturation at 95° C for 10 min. The 764 bp amplicon was visualized on a 1% w/v agarose gel. The other qPCR assays included 5 pmoles of both forward and reverse primers and 1 pmole of the correct probe in the AgPath-ID RT-PCR buffer containing nucleotides, MgCl_2_, and Taq polymerase (Applied Biosystems, Foster City USA). After an initial denaturation step of 95° C for 10 min, the total *E. coli* and Lactobacillus PCR products were amplified over 40 cycles of 95° C for 15 s and 63° C for 30 s. The annealing temperature for amplification of *Bifidobacterium* spp. was slightly higher at 66° C. Samples were amplified on a 7500 Fast PCR machine (Applied Biosystems, Foster City, USA), and the number of bacteria was determined from the standard curve for each bacterium and expressed as the number of bacteria per gram of feces. Quantification was only accepted if the PCR efficiency was between 90% and 110% and if the standard curve correlation plot had a correlation coefficient greater than 0.98.

### Statistical analyses

The statistical analyses of production data, fecal score, fecal *E. coli* excretion, and DI were performed using one-way ANOVA in SPSS (IBM SPSS, Version 24; USA) with dietary treatment as the independent variable and batch as a random factor (to account for a difference in start weight between batches of pigs). Post-hoc comparisons with the least significant difference were used to compare treatment means. Two pigs died during the study (see “Results” section) and their performance data were included for analysis in the first 2 weeks only. Two-way ANOVA was used to analyze plasma measures, fecal SCFA, fecal bacterial counts, and bacterial ratios that were measured on days 4 and d 11. One pig was removed from the plasma urea analysis due to extreme post-infection sickness.

Fecal bacterial counts for total *E. coli*, F4 *E. coli*, and *Lactobacillus* spp., and ratios for F4 *E. coli*:total *E. coli*, F4 *E. coli*:Lactobacillus, and total *E. coli:*Lactobacillus, were not normally distributed and therefore were logarithmically transformed prior to analysis. Means were then back-transformed and expressed as least-square means with 95% confidence intervals. Pen was used as the experimental unit for performance, fecal score, fecal *E. coli* excretion, and DI. Pig was used as the experimental unit (with pen as a random effect) for plasma measurements, fecal SCFA assessments, and bacterial enumeration by qPCR. Chi-squared analysis was used to compare the percentage of pigs assessed as having PWD between the different diets, and the percentage of pigs requiring therapeutic antibiotic treatment in each group. Statistical significance was accepted at *P *≤ 0.05 and *P* ≤ 0.10 was considered a trend.

## Results

One animal (control) was removed from the study prior to F4-ETEC challenge due to lameness. Two other pigs (control and HAMSA diets) died during the study due to severe inflammation in the abdominal and thoracic cavities, attributed to non-hemolytic *E. coli* (that the pigs entered the experiment with) in addition to experimental infection with F4-ETEC.

### Incidence and severity of PWD and shedding of *E. coli*

Statistically fewer (8.7%; χ^2^ = 0.035) pigs supplemented with ZnO had PWD in the 2 weeks after weaning compared to pigs fed the control or HAMSA diets. There was no difference (*P *> 0.05) in PWD incidence between the HAMSA- and control-fed pigs. Fewer (*P* = 0.016) pigs fed the ZnO diet required therapeutic antibiotic treatment for PWD compared to pigs fed the control diet, with no difference (*P *> 0.05) between HAMSA- and control-fed pigs or HAMSA and ZnO-fed pigs. The DI was lowest (*P* = 0.032) in pigs fed ZnO compared to pigs fed the other diets. There was an increase (*P* = 0.001) in the hemolytic *E. coli* scores after infection, with no difference between the treatments (*P* > 0.05) (Table 3).

**Table 3.** Effects of dietary treatment on post-weaning diarrhea (PWD), the diarrhea index (DI), and the *E. coli* score before and after inoculation, in pigs inoculated with enterotoxigenic *E. coli* (F4-ETEC) and fed different diets after weaning

Item	Treatment (T)^1^			SEM	*P* value		
	Control	ZnO	HAMSA		Day	T	D × T
% of pigs with PWD^2^	45.8^a^	8.7^b^	33.3^a^		χ^2^ = 0.035		
% of pigs treated with therapeutic antibiotic^3^	37.5^a^	4.3^b^	25^a^^,^^b^		χ^2^ = 0.016		
DI (%)^4^^,^^5^	3.37^a^	1.34^b^	2.75^a^	0.702		0.032	
*E. coli* score^6^^,^^7^							
Days 0–3 (preinoculation)	0.009	0.002	0.009	0.045	0.001	0.646	0.380
Days 5–11 (postinoculation)	1.026	0.978	0.651				

Abbreviation: SEM = standard error of the mean.

^1^Control: control diet; ZnO: control + 3,000 mg ZnO/kg; HAMSA: control + 50 g HAMSA/kg.

^2^PWD defined as pigs having a fecal consistency score of 4.

^3^Pigs assessed as having severe diarrhea (score 4) requiring treatment.

^4^The mean proportion of days with diarrhea with respect to 14 days after weaning.

^5^Data not normally distributed were logarithmically transformed and then analyzed. Means were then back-transformed.

^6^Data not normally distributed were square-root transformed. Means were then back-transformed.

^7^Agar plates were scored from 0 to 5 according to the number of streaked sections containing viable hemolytic *E. coli*, where 0 indicated no growth and 5 indicated growth out in the fifth section of the plate.

^a,^ ^b^Within a row, means without a common superscript are significantly different (*P* < 0.05).

### Performance data

There was no difference (*P *> 0.05) in live weight (LW) between treatments on days 0, 7, or 14, but there was a trend (*P* = 0.064) on day 21 for ZnO- and HAMSA-fed pigs to weigh ~ 10% more than control-fed pigs (Table 4). There was a batch effect for LW on day 21 only (*P* = 0.031), where pigs in the first batch were heavier than their counterparts in the second batch.

**Table 4.** Performance of pigs inoculated with enterotoxigenic *E. coli* (F4-ETEC) and fed different diets after weaning

Item	Treatment^1^			SEM	*P* value
	Control	ZnO	HAMSA		
LW (kg)					
d 0	6.0	6.1	6.0	0.09	0.797
d 7	6.5	7.3	7.0	0.26	0.549
d 14	7.8	8.6	8.3	0.19	0.146
d 21	9.6	10.9	11.0	0.27	0.064
ADG (g)					
d 0–7	67	97	85	9.0	0.350
d 8–14	189^a^	250^b^	259^b^	10.8	0.020
d 15–21	267^a^	323^a^^,^^b^	387^b^	16.6	0.017
d 0–21	174^a^	226^b^	245^b^	9.1	0.009
ADFI (g)					
d 0–7	111	126	113	7.1	0.587
d 8–14	265	323	325	11.9	0.063
d 15–21	404^a^	489^b^	502^b^	17.7	0.048
d 0–21	260	313	314	10.8	0.065
FCR (g/g)					
d 0–7	1.75	1.42	1.45	0.033	0.398
d 8–14	1.41	1.31	1.27	0.015	0.394
d 15–21	1.55^a^	1.52^a^	1.30^b^	0.013	0.044
d 0–21	1.50^a^	1.40^a^	1.28^b^	0.007	0.003

Abbreviation: LW = liveweight; SEM = standard error of the mean.

^1^Control: control diet; ZnO: control + 3,000 mg ZnO/kg; HAMSA: control + 50 g HAMSA/kg.

a, ^b^Within a row, means without a common superscript are significantly different (*P* < 0.05).

During days 8–14 of the study, average daily gain (ADG) was higher (*P *= 0.020) in pigs receiving ZnO and HAMSA compared to those fed the control diet, and pigs receiving the HAMSA diet had a higher ADG than those fed the control diet for days 15–21 (*P *= 0.017). The ADG for pigs fed ZnO- and HAMSA-supplemented diets were not different (*P *> 0.05) throughout the study. Across the entire 21-day period, pigs fed the ZnO- and HAMSA-supplemented diets had a higher (*P* = 0.009) ADG than those receiving the control diet (Table 4). Pigs in the first batch grew faster than pigs in the second batch on days 15–21 (*P* = 0.043) and for the overall study (*P* = 0.041).

There was a trend (*P* = 0.063) for the average daily feed intake (ADFI) of the ZnO- and HAMSA-fed pigs to be higher than control-fed pigs during days 8–14, and this became significant (*P* = 0.048) for days 15–21. Overall, there was a trend (*P* = 0.065) for pigs fed the ZnO diet or HAMSA diet to eat ~ 20% more than those fed the control diet but without there being a difference (*P *> 0.05) between ZnO- and HAMSA-fed pigs (Table 4). Pigs in the first batch ate more feed than pigs in the second batch in the first week only (*P* = 0.027).

The feed conversion ratio (FCR) was lower (*P *= 0.044) in pigs fed the HAMSA diet in days 15–21 only compared to pigs fed the control or ZnO diets. Over the 21-day period, pigs receiving the HAMSA diet had a lower FCR (*P* = 0.003) than those fed the control or ZnO diets (Table 4). There was no effect of batch on FCR in any week or for the overall study (*P* > 0.05).

### The SCFA concentrations and pH

The total SCFA concentration, and the concentrations of acetic acid, propionic acid, butyric acid, and valeric acid, were higher (*P *< 0.05) on day 4 than on day 11, with no (*P *> 0.05) diet differences. The molar proportion of butyric acid showed a significant day (*P* = 0.002) and treatment (*P* = 0.048) effect, with that in HAMSA-fed pigs being lower than that in pigs fed ZnO but the same as in control-fed pigs (data not shown).

Fecal pH showed statistically significant day and treatment effects but ultimately was determined by a significant interaction (*P* = 0.009) such that only the pH in control-fed pigs was lower at day 11 versus day 4 (7.02 vs. 7.79) (Table 5).

**Table 5.** Interaction means for the concentration of SCFA (mmol/L) and pH in feces both pre- and postinoculation in pigs inoculated with enterotoxigenic *E. coli* (F4-ETEC) and fed different diets after weaning

Item	Treatment (T)^1^			SEM	*P* value		
	Control	ZnO	HAMSA		Day	T	Day × T
Total SCFA							
d 4	59	84	79	3.9	<0.001	0.475	0.662
d 11	103	116	106				
Acetic acid							
d 4	33	48	45	2.3	<0.001	0.342	0.805
d 11	57	66	60				
Propionic acid							
d 4	13	18	16	1.0	<0.001	0.873	0.656
d 11	25	25	25				
Butyric acid							
d 4	5	10	7	0.6	<0.001	0.156	0.483
d 11	12	14	12				
Isobutyric acid							
d 4	2.3	2.0	2.7	0.15	0.900	0.887	0.310
d 11	2.4	2.8	2.4				
Valeric acid							
d 4	1.8	2.4	2.7	0.17	0.050	0.613	0.145
d 11	3.0	3.6	2.9				
Isovaleric acid							
d 4	3.6	3.3	4.8	0.04	0.743	0.949	0.337
d 11	3.7	4.4	3.8				
Caproic acid							
d 4	0.4	0.6	0.5	0.08	0.883	0.318	0.417
d 11	0.6	0.5	0.4				
pH							
d 4	7.49	7.31	7.73	0.07	<0.001	0.025	0.009
d 11	7.02	7.11	7.39				

Abbreviation: SEM = standard error of the mean.

^1^Control: control diet; ZnO: control + 3,000 mg ZnO/kg; HAMSA: control + 50 g HAMSA/kg.

### Quantitative real-time PCR analyses

For both sampling points, there was an average of 3.94 × 10^7^, 3.52 × 10^7^, and 5.37 × 10^5^ bacteria detected per gram of feces for total *E. coli*, *Lactobacillus* spp., and F4 *E. coli*, respectively. There was an average of 2.98 × 10^7^*Bifidobacteria* spp. detected per gram of feces overall, but due to undetectable levels of *Bifidobacteria* spp. in post-infection samples, only the pre-infection samples were able to be statistically analyzed. There was a strong trend (*P* = 0.057) for the HAMSA-fed group to have a higher *Bifidobacteria* spp. count than pigs in the ZnO-fed group and the control group (4.56 × 10^6^ vs. 3.24 × 10^4^ and 3.22 × 10^4^, respectively).

There was a main effect of treatment (*P* = 0.042) for total *E. coli* count, with a trend (*P* = 0.093) for pigs in the ZnO-fed group to have a lower total *E. coli* count than pigs fed the control diet, and for pigs fed HAMSA to have a higher (*P* = 0.016) total *E. coli* count than pigs fed the ZnO diet. Numbers of F4 *E. coli* and *Lactobacillus* spp. were unaffected by treatment or day or their interaction (*P* > 0.05), although there was a trend (*P *= 0.079) for Lactobacillus count to be higher on d 11 than d 4 (Table 6).

**Table 6.** Selected bacterial counts (expressed as number of bacteria per gram of feces) on day 4 and day 11 in pigs inoculated with enterotoxigenic *E. coli* (F4-ETEC) and fed different diets after weaning

Item	Treatment (T)^1^			SEM	*P* value		
	Control	ZnO	HAMSA		Day	T	Day × T
Total *E. coli*^2^ (# bacteria/g feces)							
d 4	1.91 × 10^7^	3.32 × 10^6^	9.77 × 10^6^	0.140	0.211	0.042	0.468
d 11	3.05 × 10^6^	9.29 × 10^5^	1.29 × 10^7^				
F4 *E. coli*^2^^,^^3^ (# bacteria/g feces)							
d 4	3.37 × 10^4^	4.20 × 10^4^	2.90 × 10^4^	0.136	0.428	0.579	0.489
d 11	1.68 × 10^5^	2.57 × 10^4^	4.93 × 10^5^				
*Lactobacillus* spp.^2^ (# bacteria/g feces)							
d 4	4.80 × 10^6^	3.88 × 10^6^	4.36 × 10^6^	0.200	0.079	0.836	0.490
d 11	4.70 × 10^6^	2.00 × 10^7^	1.97 × 10^7^				

Abbreviation: SEM = standard error of the mean.

^1^Control: control diet; ZnO: control + 3,000 mg ZnO/kg; HAMSA: control + 50 g HAMSA/kg.

^2^Data not normally distributed were logarithmically transformed. Means were back-transformed.

^3^The specific *E. coli* used for inoculation in the experiment.

The number of F4 *E. coli*:total *E. coli* increased (*P* = 0.013) after F4-ETEC inoculation in all diet groups, and a trend (interaction; *P* = 0.088) was observed for pigs fed HAMSA to have a lower ratio on d 11. No significant interactions (*P *> 0.05) between day and treatment for any of the other bacterial ratios were observed. The ratio of total *E. coli*:*Lactobacillus* spp. decreased (*P* = 0.039) following inoculation (Table 7).

**Table 7.** Ratios of selected bacterial counts on day 4 and day 11 in pigs inoculated with enterotoxigenic *E. coli* (F4-ETEC) and fed different diets after weaning

Item	Treatment (T)^1^			SEM	*P* value		
	Control	ZnO	HAMSA		Day	T	Day × T
F4 *E. coli*:total *E. coli*^2^							
d 4	0.18 (0.02–2.05)	1.26 (0.20–7.85)	0.30 (0.05–1.85)	0.182	0.013	0.192	0.088
d 11	5.51 (0.91–1.85)	2.76 (0.72–10.57)	0.38 (0.10–1.46)				
F4 *E. coli*^3^:*Lactobacillus* spp.^2^							
d 4	0.70 (0.02–27.29)	1.08 (0.07–16.52)	0.67 (0.04–10.16)	0.243	0.476	0.798	0.793
d 11	3.57 (0.14–93.97)	0.13 (0.01–1.47)	0.25 (0.02–2.86)				
*E. coli: Lactobacillus* spp.^2^							
d 4	0.26 (0.01–7.52)	0.32 (0.03–3.43)	0.67 (0.07–6.19)	0.267	0.039	0.308	0.690
d 11	0.36 (0.02–6.41)	0.11 (0.01–0.83)	0.25 (0.04–1.73)				

Abbreviation: SEM = standard error of the mean.

^1^Control: control diet; ZnO: control + 3,000 mg ZnO/kg; HAMSA: control + 50 g HAMSA/kg.

^2^Data not normally distributed were logarithmically transformed. Means were back-transformed.

^3^The specific *E. coli* used for inoculation in the experiment.

### Blood measurements

Haptoglobin concentrations increased (*P* = 0.021) between days 4 and 11, and the haptoglobin concentration was higher (*P* = 0.010) for pigs fed the control or HAMSA diets than for pigs fed the ZnO diet. The concentration of D-lactate was higher (*P* = 0.028) in all treatment groups on day 11 but was not different (*P *> 0.05) between dietary treatments. The concentration of plasma urea was lowest (*P* = 0.001) for pigs fed HAMSA and ZnO relative to pigs fed the control diet. There was a trend (*P* = 0.100) for pigs fed ZnO to have a higher TAC than pigs fed the other diets (Table 8).

**Table 8.** Interaction means for concentrations of selected blood metabolites in pigs inoculated with enterotoxigenic *E. coli* (F4-ETEC) and fed different diets after weaning

Item	Treatment (T)^1^			SEM	*P* value		
	Control	ZnO	HAMSA		Day	T	Day × T
Haptoglobin (mg/mL)							
d 4	0.92	0.72	0.88	0.060	0.021	0.010	0.117
d 11	1.31	0.65	1.30				
D-lactate (μmol)							
d 4	65.7	56.1	60.6	2.27	0.028	0.154	0.796
d 11	80.6	70.1	67.6				
Plasma urea (mmol/L)							
d 4	4.7	4.0	3.1	0.10	0.191	<0.001	0.503
d 11	5.1	4.7	3.1				
TAC (μM)							
d 4	241	246	232	3.1	0.280	0.100	0.636
d 11	231	246	227				

Abbreviation: SEM = standard error of the mean; TAC = total antioxidant capacity.

^1^Control: control diet; ZnO: control + 3,000 mg ZnO/kg; HAMSA: control + 50 g HAMSA/kg.

## Discussion

In contrast to the hypothesis proposed, feeding HAMSA did not reduce PWD or the DI compared to the ZnO-fed group, and the percentage of pigs displaying clinical diarrhea and the DI was significantly lower in pigs given ZnO compared to both other diets. This was reflected in an overall lower percentage of pigs requiring therapeutic antibiotic treatments. These data support numerous other studies indicating that pigs fed pharmacological levels of Zn after weaning typically show less diarrhea relative to when it is either not included or is included only at physiological levels (e.g., Bonetti et al., 2021; Canibe et al., 2022; Luise et al., 2024). In this regard, the level of Zn recorded in the HAMSA diet in the current study was higher than anticipated, perhaps due to cross-contamination during diet preparation, but nonetheless, was at a level insufficient for protection against F4-ETEC (Luise et al., 2024). Nevertheless, fecal *E. coli* plate scores were similar between diets and only increased by day 11, suggesting that ZnO may not act to reduce F4-ETEC excretion per se but through other mechanisms (Bonetti et al., 2021; Tang et al., 2024). Such a lack of difference between diets has been reported previously using the same F4-ETEC inoculation model (Stensland et al., 2015). In this sense, Roselli et al. (2003) suggested ZnO reduces bacterial adherence of F4-ETEC in the small intestine and blocks bacterial invasion through the prevention of increased tight junction permeability, rather than having a direct bactericidal effect. Inhibition of F4-ETEC adherence could prevent the induction of an inflammatory response in infected cells and subsequent disruption of membrane integrity, thus reducing the severity of PWD. Zhang and Gou (2009) reported that pharmacological Zn improved barrier integrity by influencing tight junction proteins to reduce intestinal permeability, a result confirmed also by Collins and Bowring (2023). Be this as it may, no significant differences in D-lactate concentration, generally considered a marker for GIT barrier function, were observed between diets in the current study. Furthermore, Hansen et al. (2022) reported no difference in D-lactate concentration after weaning in pigs fed 153, 493, 1,022, 1,601, 2,052, or 2,407 mg Zn/kg (added as ZnO).

Another possible reason for the disparity between diarrhea indices and *E. coli* shedding relates to variability between pigs in the expression of receptors in the small intestine for *E. coli* attachment (Fairbrother et al., 2005). The presence of these receptors varies between genotypes, and as such pigs used in the present study were sampled after arrival and then retrospectively screened for these receptors (Sterndale et al., 2019). It was found that ~ 73% of pigs were MUC-4 homozygote or heterozygote susceptible, and as pigs were evenly distributed across the treatments, there was no association between susceptibility and dietary treatment.

Dietary supplementation with HAMSA has been shown previously to improve GIT health outcomes in rodent and human studies of acute colonic bacterial infections. In a whole-gut perfused rat model of cholera-toxin-induced diarrhea, HAMSA promoted large bowel fluid and electrolyte uptake more effectively than nonacylated starches, and butyrylated and propionylated high-amylose maize starches (Clarke et al., 2011a). In humans, HAMSA added to an oral rehydration solution shortened the duration of diarrhea experienced during acute infectious gastroenteritis (Pal et al., 2013), which may be the result of increased large bowel SCFA promoting fluid and electrolyte uptake in the colon. In models of colonic infections, HAMSA improved mucosal barrier function in mice infected with enterohemorrhagic *E. coli* (Fukuda et al., 2011) and promoted favorable changes in the GIT microbiota, modulated inflammatory responses and affected expression of pathogen virulence genes in mice infected with *C. rodentium* (Yap et al., 2021). These effects of HAMSA were largely mediated in the large bowel where most of the esterified acetate is released by the microbiota. In the current study, HAMSA may not have been effective at reducing PWD because F4-ETEC adheres to specific receptors in the small intestine where limited amounts of esterified acetate would be released, although the G-protein coupled receptors (GPR; GPR43, and GPR41) to which acetate binds are expressed in both the colon and the enteroendocrine cells of the ileum (Nøhr et al., 2013). In contrast, feeding raw potato RS (RS type 2) to pigs improved several enteric disease outcomes during *Salmonella typhimurium*, which involves disease in both the ileum and colon, and F4-ETEC infections (reviewed by Regassa and Nyachoti, 2018; Tan et al., 2021), suggesting differences between RS types and their impacts on these diseases of pigs. Martin et al. (1998) remarked that the physical structure of RS is a major factor contributing to its effects on SCFA production, the microbiome in the cecum and colon, and subsequent physiological impacts.

Alternatively, or complementarily, the HAMSA inclusion rate of 50 g/kg used in the current study was insufficient to elicit any GIT responses. The lack of any increases in fecal SCFA concentration or acetate concentration, or decreased fecal pH, suggests that this level of dietary inclusion may not have exceeded the microbial fermentative, and (or) GIT absorptive capacities, of acetate. Mariño et al. (2017) fed a diet containing 150 g HAMSA/kg to mice and observed a significant increase in fecal acetate concentration. In a review in pigs, albeit with RS-2, Metzler-Zebeli et al. (2019) concluded that dietary inclusion rates of 100–150 g/kg were required before any appreciable impacts on indices of GIT function, including lowered digesta pH, were observed. A diet with 50 g HAMSA/kg increased the concentration of acetate in the ileum and cecum of broilers, but fecal concentrations were not measured (M’Sadeq et al., 2015). Moreover, fecal sampling, as was conducted in the present study, may be less sensitive compared to cecal or colonic samples to changes in fermentation patterns, and hence more specific effects of the diets on SCFA production may have inadvertently been missed.

Total *E. coli* counts were lowest for pigs in the ZnO-fed group, but there was no difference between treatments or before and after inoculation for F4 *E. coli* counts. This supports several other studies (Stensland et al., 2015; Collins and Bowring, 2023) and reinforces the variety of actions pharmacological Zn elicits to ease the post-weaning transition and reduce PWD, as discussed previously. Recently, Ortiz Sanjuán et al. (2024) compared the effects of in-feed antibiotics and therapeutic doses of ZnO with non-medicated animals on the development of the post-weaning microbiota on four commercial pig farms. Both antibiotics and ZnO prevented *E. coli* overgrowth in healthy and diarrheic samples at 7 days after weaning, by modulating the transition of species and functional richness, diversity, and composition of the microbiota.

The number of F4-ETEC and *Lactobacillus* spp. observed in all groups both pre- and post-infection on days 4 and 11 were similar, a finding that contrasts with Højberg et al. (2005) who found an increase in the number of coliforms and a decrease in the number of *Lactobacillus* spp. in pigs fed a ZnO-supplemented diet. It would therefore have been expected that the lower count of *E. coli* in the ZnO treatment group may have been accompanied by an increased presence of *Lactobacillus* spp., but this was not found in the present experiment. In this regard, and although feeding HAMSA did not significantly alter total populations or ratios of bacterial populations, there was a strong statistical trend for the HAMSA-fed pigs to have a higher *Bifidobacteria* spp. count than pigs in the ZnO and control groups before inoculation, and for there to be a lower F4 *E. coli*:total *E. coli* ratio following inoculation. These data suggest supplementation with HAMSA resulted in some favorable changes to the microbiota similar to feeding pigs other RS types (Fouhse et al., 2015), albeit there being no difference in diarrhea outcomes. In human infants, Gopalsamy et al. (2019) found that fecal inocula, whether for pre-weaning or at-weaning infants, was able to utilize HAMSA as a potential substrate and increased Shannon’s diversity index, selectively stimulated *Bifidobacterium* copy numbers, and increased the Bacteroidetes:Firmicutes ratio.

The performance of pigs fed ZnO and HAMSA was similar across the 21-day period and generally higher than that of pigs fed the control diet. This is consistent with many studies (e.g., Bonetti et al., 2021; Canibe et al., 2022; Luise et al., 2024) reporting improved production in pigs fed pharmacological levels of Zn, especially in the first ~ 14 days after weaning. A noteworthy finding in this study was the significantly lower FCR in HAMSA-fed pigs in the third week after weaning, and overall, relative to both other diet groups. Growing pigs (~ 40 kg) fed high-amylose maize starch showed improved feed conversion efficiency, although because the efficiency of feed used for carcass gain did not differ to pigs fed a lower RS form of maize starch, the difference in feed efficiency was determined by the non-carcass fraction, predominately the weight of the GIT (van Erp et al., 2020). The weight of the GIT was not assessed in the current study. As an alternate, or indeed corresponding, explanation, Gardiner et al. (2020) commented that bacterial carbohydrate and (or) lipid metabolism pathways are generally enriched in the large bowel of pigs with better feed efficiency. These authors remarked that bacterial taxa involved in nutrient processing and energy harvest, as well as those associated with anti-inflammatory effects and improved GIT health, were typically enriched in more feed-efficient pigs, while potential pathogens were less abundant. This contention could not be confirmed in the current study, but it is tempting to speculate that changes to the microbiota caused by feeding HAMSA, as shown in other studies, may have been responsible for the improved FCR observed. However, whether this effect is limited to pigs recovering from an enteric infection, or applies to healthy growing pigs after weaning, could not be ascertained.

In response to infection or other injury, the immune system will usually commence the acute phase response, secreting pro-inflammatory cytokines that act as messengers between the site of infection and the hepatocytes that synthesize the acute phase proteins (APP) (Petersen et al., 2004). Porcine haptoglobin, a positive APP, has been found to increase following experimental and natural infections (Dritz et al., 1996; Petersen et al., 2002), which concurs with the current findings where haptoglobin concentrations were higher on day 11. The lower haptoglobin concentration in pigs fed ZnO likely reflects the lower proportion of these pigs experiencing diarrhea, and therefore a smaller acute phase response to infection. There is also evidence of anti-inflammatory effects of ZnO (Bergeron et al., 2017) with this reduction being consistent with that response, although in a previous study in this laboratory with F4-ETEC inoculation, no decrease in haptoglobin concentration was observed with feeding ZnO (Stensland et al., 2015). The trend observed in this study is supported by a study in which ZnO-fed commercial pigs had improved antioxidant status as measured by malondialdehyde rather than TAC (Bergeron and Guay, 2019).

Increasing levels of RS can cause a lower apparent ileal and total tract digestibility of nutrients and energy, predominately associated with changes in the rate of nutrient absorption or with increased endogenous secretions (Gerritts et al., 2012). Accordingly, fermentation of RS can reduce the apparent fecal digestibility of crude protein while increasing fecal N excretion at the expense of urinary N, due to enhanced excretion of microbial protein (Sun et al., 2006). The significantly lower plasma urea concentration in pigs fed HAMSA in this study may be the result of increased microbial activity due to the supply of dietary RS providing fermentative substrate to the large bowel microbiota rather than any differences in Zn concentrations per se between the ZnO and HAMSA diets. Pieper et al. (2012) reported no differences in digesta ammonia concentrations, which after absorption is converted to urea, in piglets offered diets formulated to contain 50, 150, 250, 1,000, and 2,500 mg Zn/kg (as ZnO).

## Conclusion

This study demonstrated that supplementing piglets with dietary ZnO, but not HAMSA, reduced the incidence of PWD in piglets inoculated with a strain of F4-ETEC. Both ZnO- and HAMSA-fed pigs displayed increased ADG and ADFI compared to control piglets fed no supplements. The FCR was significantly improved in the third week, and in the overall study, in pigs fed HAMSA compared to control and ZnO-supplemented piglets suggesting that HAMSA may benefit the growth and productivity of pigs following an enteric disease challenge. The lack of any significant effects in fecal total SCFA and acetate concentrations suggests that the inclusion rate of HAMSA in this study may have been insufficient to elicit positive diarrhea outcomes.

## Abbreviations

ADFI: average daily feed intake
ADG: average daily gain
DI: diarrhea index
ETEC: enterotoxigenic *Escherichia coli* (*E. coli*)
FCR: feed conversion ratio
GIT: gastrointestinal tract
HAMSA: acetylated high-amylose maize starch
PWD: post-weaning diarrhea
RS: resistant starch
SCFA: short-chain fatty acid
SEM: standard error of the mean
SID: standardized ileal digestible
ZnO: zinc oxide
